# The effect of composite enzyme catalysis whey protein cross‐linking on filtration performance

**DOI:** 10.1002/fsn3.2265

**Published:** 2021-03-30

**Authors:** Wang Wen‐qiong, Zhou Ji‐yang, Yu Qian, Li Jianju

**Affiliations:** ^1^ College of Food Science and Engineering Yangzhou University Yangzhou China; ^2^ Jiangsu Key Laboratory of Dairy Biotechnology and Safety Control Yangzhou University Yangzhou China; ^3^ Weiwei Food and Beverage Co., Ltd Xuzhou China

**Keywords:** cross‐linking, enzyme, fouling mechanism, ultrafiltration, whey protein

## Abstract

In this study, enzymatic cross‐linked whey protein coupling ultrafiltration was used to reduce membrane fouling and increase whey protein recovery rate. The filtration efficiency and protein interaction with the membrane surface were investigated. The results showed that the protein recovery rate and relative flux of transglutaminase catalysis protein followed by tyrosinase each increased by approximately 30% during ultrafiltration. The total membrane resistance was reduced by approximately 20%. The shape of the transglutaminase and tyrosinase cross‐linked protein had somewhat spherical and cylindrical structure similar to an elongated shape based on fluorescence microscopy imaging, which indicated membrane resistance reduction. Fluorescence excitation–emission matrix spectroscopy (EEM) showed that the permeation peak intensities of transglutaminase followed by tyrosinase catalysis protein decreased sharply in the tryptophan and aromatic‐like protein fields, indicating that most protein was rejected after ultrafiltration. The repulsive interaction energy was increased between the cross‐linked proteins and membrane based on extended Derjaguin–Landau–Verwey–Overbeek (XDLVO) analysis.

## Graphical abstract

The efficiency of enzyme cross‐linked whey protein coupled with ultrafiltration was evaluated. Relationship between membrane resistance and different cross‐linked proteins were investigated. Protein and membrane interactions were used to help revealing membrane fouling mechanism.

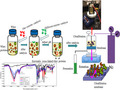

## 1 INTRODUCTION

Whey is the primary by‐product in the cheese industry and can cause serious pollution if it is discharged without any treatment. Low protein concentration and the substantial amount of treatments required are the current main problems. Therefore, increasing membrane recovery efficiency and obtaining functional proteins have vital significance. Currently, ultrafiltration is utilized to fractionate the protein from whey to avoid environmental pollution and waste treatment costs (Artemi et al., 2020). However, membrane fouling caused by long‐term membrane filtration operations causes a substantial problem in membrane recovery. There are some methods that can reduce protein fouling such as diafiltration (Hartinger & Kulozik, 2020), ultrasound (Aghapour Aktij et al., 2020), and electric field (Corbatón‐Báguena et al., 2016) and magnetic field treatments (Kyle, 2006). Reducing the diafiltration steps were also important (Hartinger & Kulozik, 2020). The membrane filtration is very complex and many factors limit membrane operation, especially long‐term operation which is very difficult to control. Therefore, reducing membrane fouling and increasing the protein recovery rate are still issues that need to be resolved.

There are three reasons for membrane fouling during the whey ultrafiltration process. First, the membrane fouling phenomenon is due to the protein–protein and protein–membrane interaction forces and depends on different factors such as pH, temperature and composition of the feed solution, characteristics of the membrane (pore size and material), and the operating conditions (transmembrane pressure and cross flow velocity). Second, hydrophobicity and surface charge are also two physicochemical properties that exert an important influence on the extent of adsorption in aqueous systems. They counteract the interactions of hydrophobic attraction and electrostatic repulsion, respectively (Wang et al., 2020). There are several basic aspects of membrane fouling: (a) the effect of membrane hydrophobicity, (b) effect of membrane surface charge, (c) the effect of protein hydrophobicity, (d) the effect of protein surface charge, and (e) the distribution of membrane pore and solute sizes (Wen‐Qiong et al., 2019).

In this study, double enzymes (one enzyme catalysis followed by catalysis by a different enzyme) were used to catalyze whey protein cross‐linking coupled with ultrafiltration which attempted to induce more protein cross‐linking with membrane fouling decreased and increased protein recovery rate. Four polymerases (horseradish peroxidase (Saricay et al., 2017), tyrosinase (EC 1.14.18.1) (Li et al., 2020), laccases (EC 1.10.3.2) (Jiang et al., 2017), and transglutaminase (EC 2.3.2.13) (Wang et al., 2018) were used to catalyze whey protein cross‐linking. The four enzymes used in the research had been used for catalysis whey protein cross‐linking in the literatures. The enzymatic cross‐linked whey protein could improve the property of the protein such as viscosity, thermal stability, water holding capacity, foaming, and emulsion.

However, the study of double enzyme catalysis in whey protein cross‐linking followed by ultrafiltration is limited, especially in cheese whey. With an improved understanding of the fouling mechanism, whey protein has received more attention because of membrane fouling. In this study, we attempt to use double enzymes to catalyze protein cross‐linking in whey following by ultrafiltration in order to increase whey protein filtration efficiency and decrease protein interactions with the membrane surface. The whey protein recovery rate, rejection rate, volumetric concentration factor (VCF), relative flux, and fluorescence excitation–emission matrix spectroscopy (EEM) were used to investigate the membrane filtration efficiency after enzyme treatment. In addition, fluorescence microscopy was also used to image the shape changes of the cross‐linked protein catalyzed by double enzymes and reveal the relationship to membrane resistance. In this study, XDLVO models which have been applied to describe the combined effect of membrane and foulant hydrophobicity and surface charge on adsorptive fouling during filtration were used to characterize between protein–protein and protein–membrane interaction including the total interaction energy per unit area (E) of the particle surface in terms of Lifshitz–van der Waals force (LW), electrostatic force (EL) energy, and acid–base (AB) interaction energy (Ding et al., 2013).

## 2 MATERIALS AND METHODS

### 2.1 Materials

Polyethersulfone membrane (PES) with a molecular weight cutoff of 10 kDa was obtained from Sepro Co. America. Chemical Co. Transglutaminase (EC 2.3.1.13) (1,000 U/g) was supplied by Taixing Yiming biological Co., Ltd. (Jiangsu, China). Horseradish peroxidase (300 U/g, CAS: 1112A053), tyrosinase (570 U/g, CAS: 78830), and laccase (3,000 U/g, CAS: 80498–15–3) were purchased from Solarbio. Caffeic acid, ferulic, and DL‐Dithiothreitol (DTT) were obtained from Aladdin company. Whey was obtained from the production of cheddar cheese from skim milk in our laboratory. The whey contained 0.4% (m/v) protein, 6% (m/v) lactose, 0.04% (m/v) lactate, and 0.03% (m/v) calcium. The original pH was 4.6, and it was stored in a 4°C refrigerator.

### 2.2 Methods

#### 2.2.1 Experimental procedure

The enzyme catalysis was based on the previous literature. In the horseradish peroxidase catalysis condition, 100 U/ml of enzyme was added at 40°C and mixed for 2 hr at pH 5.0 with H_2_O_2_ 1 mmol/L (Heijnis et al., 2010). In the tyrosinase catalysis conditions, 450 U/ml enzyme was added at 50°C and mixed for 3 hr at pH 7.0 with 1 mmol/L of caffeic acid (Thalmann & Lötzbeyer, 2002). In the laccase catalysis condition, 9 U/ml of enzyme was added at 40°C and mixed for 2 hr at pH 4.5 with 5 mol/L ferulic acid (Jus et al., 2011). In the transglutaminase catalysis conditions, 40 U/ml of enzyme was added at 40°C and mixed for 1 hr at pH 5.0 with 20 mmol/L DTT (DL‐Dithiothreitol) (Færgemand et al., 1997). The enzyme catalytic effect was evaluated. The effects of sequential additional enzymes were evaluated (e.g., Laccase treatment followed by TG catalysis was labeled “Laccase + TG,” Peroxidase treatment followed by TG catalysis was labeled “Peroxidase + TG,” Tyrosinase catalysis followed by TG catalysis was labeled “Tyrosinase + TG,” TG treatment followed by Tyrosinase catalysis was labeled “TG + Tyrosinase,” TG treatment followed by Laccase catalysis was labeled “TG + Laccase,” TG treatment followed by Peroxidase catalysis was labeled “TG + Peroxidase”). 2 mol/L HCl and 2 mol/L NaOH were used to adjust the pH of whey solution. Then, the double enzyme‐treated samples were poured separately into a Millipore dead‐end UF system. This system had total volume of 40 ml. The stirring speed was 300 r/min. The filtration began with a transmembrane pressure (TMP) of 0.15 MPa.

#### 2.2.2 Ultrafiltration efficiency evaluation

The whey protein concentration was determined using the Lowry protein assay method (Metsämuuronen et al., 2011).

(1) $$\text{Whey}\;\text{protein}\;\text{recovery}\;\text{rate}=\left(C_{\text{retentate}}\;V_{\text{retentate}}\right)/\left(C_{\text{feed}}\;V_{\text{feed}}\right)\times 100\%$$

(2) $$\text{Whey}\;\text{protein}\;\text{rejection}\;\text{rate}=\left(1‐C_{\text{permeate}}/C_{\text{feed}}\right)\times 100\%$$

where *C*_feed_, *C*_permeate,_ and *C*_retentate_ are the protein concentrations of the feed, permeate, and retentate, respectively. *V*_retentate_ and *V*_feed_ (ml) are the volumes of the retentate and feed (He et al., 2011).

At a determined volumetric concentration factor (VCF), the samples of permeate and concentrate were collected. The VCF was defined as:

(3) $$\text{VCF}\;=\;V_{i}/\left(V_{i}‐V_{p}\right)$$

where *V_i_* was the initial volume of the feed, and *V_p_* was the permeate volume (Macedo et al., 2012).

#### 2.2.3 Permeate flux measurement

Permeate flux was calculated using the following equation:

(4) $$J\;=\;\Delta V/\left(A\Delta t\right)$$

(5) $$\text{Relative}\;\text{flux}\;=\;J/J_{0}$$

where *V* (ml) was the volume of permeation at different ultrafiltration times, *A* (cm^−2^) was the membrane surface area, Δ*t* (min) was the time between two mass measurements, and *J*_0_ was the initial pure water flux (Miller et al., 2014).

#### 2.2.4 Membrane resistance measurement

Membrane resistance was calculated according to the Darcy equation:

(6) $$\text{Filtration}\;\text{flux:}\;J=\frac{\Delta P}{\mu R_{t}}\;R_{t}=\frac{\Delta P}{\mu J}\;J_{w}=\frac{\Delta P}{\mu_{w}R_{m}}\;R_{m}=\frac{\Delta P}{\mu_{w}J_{w}}$$

$$\text{Total}\;\text{membrane}\;\text{resistance}:\;R_{t}=R_{m}+R_{c}+R_{p}$$

(7) $$R_{p}+R_{m}=R_{w}\;R_{p}=R_{w}‐R_{m}\;R_{c}=R_{t}‐R_{m}‐R_{p}$$

where *J* was the sample permeate flux, Δ*P* was the transmembrane pressure, *μ* is the sample dynamic viscosity, *J_w_* and *μ_w_* were water viscosity and pure water fux, *R_t_* was the total resistance, *R_m_* was the membrane hydraulic resistance, *R_w_* was the sum of *R_m_* and *R_p_* which assumed that all foulant on the membrane surface can be removed by water surface washing without affecting membrane pore blockage, *R_w_* was calculated after membrane surface washing, *R_p_* was the resistance due to pore blocking, and *R_c_* was the resistance due to cake formation (Listiarini et al., 2009).

#### 2.2.5 Fluorescence microscopy imaging whey protein and cross‐linking protein

The whey protein and enzyme catalysis cross‐linking protein were dyed by Rhodamine B (13 μmol/L), and the dyed protein was then dropped on to the cover glass. The proteins were imaged using a Zeiss Axio Vert A1 fluorescence microscope with DsRed (Carl Zeiss Ltd.) (Ugwu et al., 2016).

#### 2.2.6 Fluorescence excitation–emission matrix spectroscopy (EEM)

Emission matrix spectroscopy was measured in a 1 cm quartz cuvette (4 ml volume) using a Hitachi Fluorescence Spectrophotometer (F‐7000) equipped with FL Solutions 2.1 for data processing. Fluorescence EEMs were evaluated for excitation wavelengths of 200–450 nm at 5 nm increments across an emission range of 250–550 nm at 1 nm intervals (Hambly et al., 2015).

#### 2.2.7 Scanning electronic microscopy (SEM)

The surfaces of membranes before and after filtration experiments were scanned by a scanning electron microscope (S‐4800ⅡSEM). A piece was cut from the membranes after filtration. The membranes were dried at room temperature and coated with gold prior to SEM observation.

#### 2.2.8 Fourier transform infrared spectroscopy

The membrane surface of filtered whey protein and enzyme‐catalyzed whey protein before and after filtration were analyzed by FTIR (Varian). Fourier transform infrared spectroscopy (FTIR) spectra were recorded in ATR mode on a Varian Cary 610/670 FTIR spectrometer, using the Turbo mode of the Ever Glo infrared source. Thirty six scans were made with a selected resolution of 8 cm^−1^.

#### 2.2.9 Derjaguin–Landau–Verwey–Overbeek modeling

Contact angles were used to calculate the surface tension components (*γ* ^LW^, *γ* ^+^, and *γ* ^−^) of the glass surface (*s*) using the Young–Dupre Equation (8), where *γ* ^LW^ is the van der Waals contribution, *γ* ^+^ is the electron–acceptor contribution, and *γ* ^−^ is the electron donor contribution (Bower et al., 2010):

(8) $$\left(1+\cos\theta \right)\gamma_{l}^{\text{TOT}}=2\left(\sqrt{\gamma_{s}^{\text{LW}}\gamma_{l}^{\text{LW}}}+\sqrt{\gamma_{s}^{+}\gamma_{l}^{‐}}+\sqrt{\gamma_{s}^{‐}\gamma_{l}^{+}}\right)$$

The subscripts *s* and l represent the solid surface and the liquid, respectively. The total interaction energy includes Lifshitz–van der Waals (LW), Lewis acid–base (AB) interactions, and electrostatic (EL) forces interaction energy. Δ*G* ^LW^(*h*), Δ*G* ^AB^(*h*), and Δ*G* ^EL^(*h*) are given by:

(9) $$\Delta G_{123}^{\text{LW}}\left(h\right)=‐\frac{A}{12\pi h^{2}}\;A=‐12\pi h_{0}^{2}\;\Delta G^{\text{LW}}$$

(10) $$\Delta G_{123}^{\text{EL}}\left(h\right)=\varepsilon \varepsilon_{0}k\xi_{f}\xi_{m}\left[\frac{\xi_{f}^{2}+\xi_{m}^{2}}{2\xi_{f}\xi_{m}}\;\left(1‐\coth\text{kh}\right)+\frac{1}{\sinh\;\text{kh}}\right]\;\frac{1}{k}=\sqrt{\varepsilon_{0}\varepsilon \;R_{g}T/\left(2F^{2}\;I_{s}\right)}$$

(11) $$\Delta G_{123}^{\text{AB}}\left(h\right)=\Delta G_{h_{0}}^{\text{AB}}\exp\;\left(\frac{h_{0}‐h}{\lambda }\right)$$

(12) $$\Delta G=\Delta G^{\text{LW}}+\Delta G^{\text{AB}}+\Delta G^{\text{EL}}$$

where *h* is the separation distance between two differential surface elements. The individual interaction energy per unit area at *h*_0_ was obtained by:

(13) $$\Delta G_{h_{0}}^{\text{LW}}=2\left(\sqrt{\gamma_{m}^{\text{LW}}}‐\sqrt{\gamma_{w}^{\text{LW}}}\right)\left(\sqrt{\gamma_{f}^{\text{LW}}}‐\sqrt{\gamma_{w}^{\text{LW}}}\right)$$

(14) $$\begin{array}{c} \Delta G_{h_{0}}^{\text{AB}}=2\sqrt{\gamma_{w}^{+}}\;\left(\sqrt{\gamma_{f}^{‐}}+\sqrt{\gamma_{m}^{‐}}‐\sqrt{\gamma_{w}^{‐}}\right)+2\sqrt{\gamma_{w}^{‐}}\;\left(\sqrt{\gamma_{f}^{+}}+\sqrt{\gamma_{m}^{+}}‐\sqrt{\gamma_{w}^{+}}\right)\\ ‐2\;(\sqrt{\gamma_{m}^{+}\gamma_{f}^{‐}}‐\sqrt{\gamma_{m}^{‐}\gamma_{f}^{+}}) \end{array}$$

(15) $$\Delta G_{h_{0}}^{\text{EL}}=\frac{\varepsilon \varepsilon_{0}\kappa }{2}\;\left(\xi_{1}^{2}+\xi_{3}^{2}\right)\;\left[1‐\coth\;\left({\text{kh}}_{0}\right)+\frac{2\xi_{1}\xi_{3}}{\xi_{1}^{2}+\xi_{3}^{2}}\;\text{csch}\;\left({\text{kh}}_{0}\right)\right]$$

Δ*G*_FM_ represents the energy needed for the adhesion of the surfaces of the foulant (*F*) and membrane (*M*) when both are immersed in water. Δ*G*_FF_ provides an indication of protein–protein interactions. The free energy of electrostatic interaction was derived (specifically, *ξ*_3_ is quantified by the zeta potential of the foulant, and *ξ*_1_ quantifies by the PES membrane), *ε* represents the dielectric constant of the water, *ε*_0_ represents vacuum permittivity, and 1/*κ* represents the Debye length. Equation (11) can be re‐expressed as (Zamani et al., 2016):

(16) $$\begin{array}{cc} \Delta G_{\text{FM}} & =2\;\left[\begin{array}{c} \sqrt{\gamma_{F}^{\text{LW}}\gamma_{M}^{\text{LW}}}+\sqrt{\gamma_{M}^{\text{LW}}\gamma_{M}^{\text{LW}}}‐\sqrt{\gamma_{F}^{\text{LW}}\gamma_{M}^{\text{LW}}}‐\gamma_{W}^{\text{LW}}+\sqrt{\gamma_{W}^{+}}\;\left(\sqrt{\gamma_{F}^{‐}}+\sqrt{\gamma_{M}^{‐}}‐\sqrt{\gamma_{W}^{‐}}\right)\\ +\sqrt{\gamma_{W}^{‐}}\;\left(\sqrt{\gamma_{F}^{+}}+\sqrt{\gamma_{M}^{+}}‐\sqrt{\gamma_{W}^{+}}\right)‐\sqrt{\gamma_{F}^{+}\gamma_{M}^{‐}}‐\sqrt{\gamma_{F}^{‐}\gamma_{M}^{+}} \end{array}\right]\;\\ & \;+\;\frac{\varepsilon \varepsilon_{0}\kappa }{2}\left(\xi_{1}^{2}+\xi_{3}^{2}\right)\;\left[1‐\coth\left({\text{kh}}_{0}\right)+\frac{2\xi_{1}\xi_{3}}{\xi_{1}^{2}+\xi_{3}^{2}}\text{csch}\left({\text{kh}}_{0}\right)\right] \end{array}$$

(17) $$\begin{array}{cc} \Delta G_{\text{FF}} & =‐2\;{\left(\sqrt{\gamma_{F}^{\text{LW}}}‐\sqrt{\gamma_{w}^{\text{LW}}}\right)}^{2}‐4\;\left[\sqrt{\gamma_{w}^{+}\gamma_{w}^{‐}}+\sqrt{\gamma_{F}^{+}\gamma_{F}^{‐}}‐\sqrt{\gamma_{F}^{+}\gamma_{w}^{‐}}‐\sqrt{\gamma_{w}^{+}\gamma_{F}^{‐}}\right]\\ & \;+0.5\;\frac{\varepsilon \varepsilon_{0}\kappa }{2}\;\left(\xi_{1}^{2}+\xi_{3}^{2}\right)\;\left[1‐\coth\;\left({\text{kh}}_{0}\right)+\frac{2\xi_{1}\xi_{3}}{\xi_{1}^{2}+\xi_{3}^{2}}\text{csch}\;\left({\text{kh}}_{0}\right)\right] \end{array}$$

#### 2.2.10 Particle size measurements

The size of enzymatic catalysis whey protein cross‐linking was determined by DLS using a Zetasizer Nano ZS from Malvern Instruments (Kinexus, Malvern Instruments Ltd.).

### 2.3 Statistical analysis

All examinations were repeated three times. Collected data are expressed as the mean ± standard deviation (*SD*). Analysis of variance (ANOVA) was performed, and means comparisons were carried out by Student–Newman–Keuls’ tests. A value of *p* < .05 was considered significant. Data were analyzed by using a statistical software package (SPSS for Windows, 11.5, 2002, SPSS Inc.).

## 3 RESULTS AND DISCUSSION

### 3.1 The effect of enzymatic cross‐linked whey protein on the ultrafiltration process

In our previous study, TG catalysis of whey protein coupled with ultrafiltration could increase membrane filtration efficiency (Wen‐Qiong et al., 2017). In this study, double enzyme catalysis was used to cause protein cross‐linking, which was expected to reduce membrane blockage and increase the protein recovery rate further. The whey protein was catalyzed by TG and then catalyzed by tyrosinase, laccase, and peroxidase. The tyrosinase, laccase, and peroxidase catalysis separately followed by TG were also investigated. The whey protein recovery rate and relative flux changes were investigated to evaluate the double enzyme catalysis coupling filtration efficiency.

**FIGURE 1:**
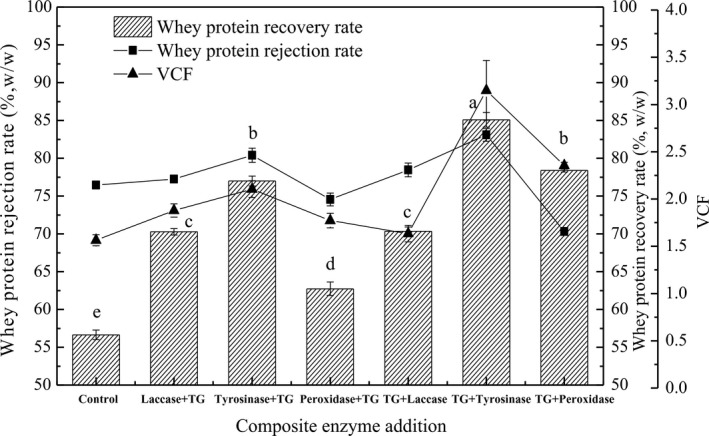
Whey protein recovery rate,rejection rate and volumetric concentration factor (VCF) of enzyme aggregated proteins coupling ultrafiltration including Laccase + TG, Peroxidase + TG, Tyrosinase + TG, TG + Tyrosinase, TG + Laccase and TG + Peroxidase

As shown in Figure 1, the whey protein rejection rate was 89% by double enzyme (TG + tyrosinase) catalysis which was higher than that of other conditions. The whey protein recovery rate could reach 84%. This indicated that little protein adsorbed on membrane surface or pores. As shown in Figure 5 (e1 and e2), there was some proteins distribution on the membrane surface which was lower than the other samples. This was due to most of the protein washed away by the retention after the filtration. However, the membrane pore blockage was difficult to wash off for protein, so the membrane pore resistance was similar to control, as shown in Figure 4(a). The VCF of the TG + tyrosinase enzyme‐catalyzed whey during filtration was also higher than the other samples, which indicated that the level of membrane fouling decreased. The whey protein recovery rates of the tyrosinase + TG and TG + laccase catalysis samples were not significant and were lower than that of the TG + tyrosinase catalysis sample by approximately 10%. This may be the cross‐linked protein was deposited on the membrane surface and adsorbed on the membrane surface. The whey protein recovery rate of the peroxidase + TG catalytic coupling UF sample was lower than that of the TG + tyrosinase enzyme catalysis sample by approximately 20%. This indicated that the peroxidase catalysis followed by TG had little effect on the membrane filtration process. These rates were mainly to proteins aggregated after enzyme catalysis. Therefore, TG followed by tyrosinase catalyzed whey protein cross‐linking could increase the membrane ultrafiltration efficiency.

### 3.2 Filtration flux analysis of enzymatic cross‐linked whey protein coupled with ultrafiltration

**FIGURE 2:**
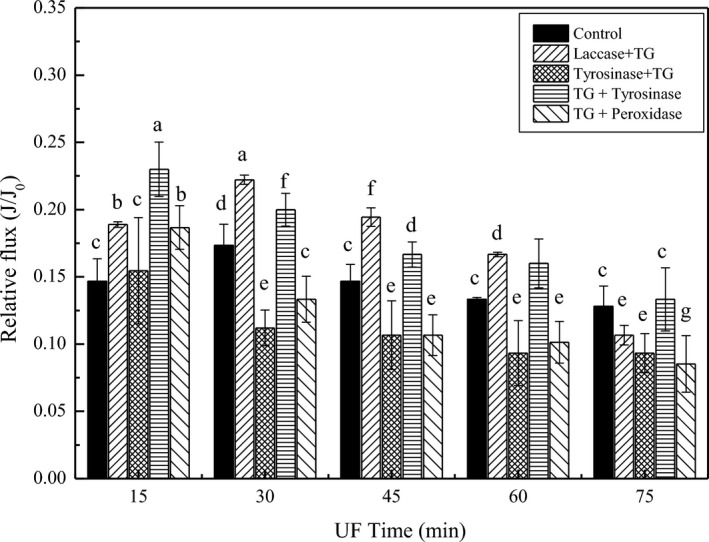
Relative flux changes of double enzyme catalysis whey protein cross‐linking including whey (Control), Laccases + transglutaminase (TG), Tyrosinase + TG, TG + Tyrosinase and TG + Peroxidase followed by ultrafiltration

As shown in Figure 2, the relative flux of tyrosinase + TG and TG + laccase catalyzed whey decreased with increased ultrafiltration time, which were even lower than that of the control. Ultrafiltration efficiency could not be improved even when protein was cross‐linked into bigger particles. This result occurred mainly because large proteins were deposited on the membrane with cake resistance formation and the membrane flux decreased. The relative flux of the laccase + TG catalysis sample was higher than that of the TG + tyrosinase catalysis sample during the 30‐ to 60‐min time period (*p* < .05), but decreased after 75 min. However, the protein recovery rate of laccase + TG catalytic whey was lower than that of the TG + tyrosinase catalyzed sample (*p* < .05), as shown in Figure 2. This difference may be due to the permeation of small proteins. The relative membrane flux of the TG + tyrosinase catalyzed whey coupled with ultrafiltration was highest of all in the initial 15 min and slightly decreased as filtration time increased. Additionally, the TG + tyrosinase catalyzed sample had the highest protein recovery rate. Therefore, TG catalysis followed by tyrosinase catalysis whey protein cross‐linking could increase relative flux during the ultrafiltration process. Although the four enzymes could catalyze whey protein cross‐linking, the relative flux levels were not at all increased compared to the control. It was assumed that many large proteins were deposited on the membrane surface forming tight cake resistance under transmembrane pressure, which made membrane filtration difficult (Haribabu et al., 2020). In this study, the TG + tyrosinase catalyzed whey protein cross‐linking could increase the membrane filtration efficiency.

### 3.3 Emission matrix spectroscopy spectra of permeation from enzymatic cross‐linked whey protein coupled with ultrafiltration

**FIGURE 3:**
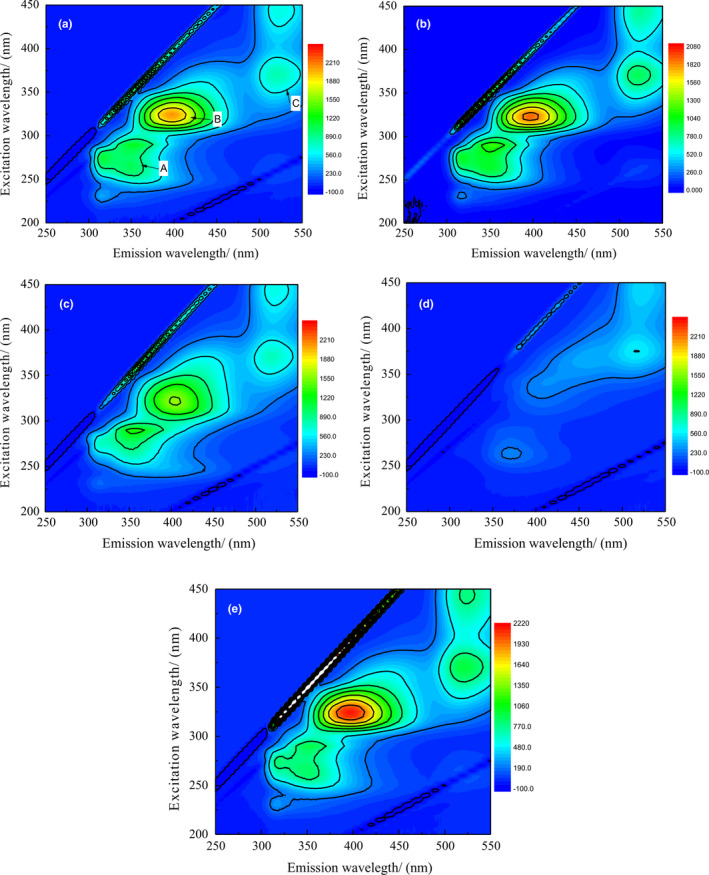
Excitation‐emission matrix spectroscopy (EEM) spectra of the ultrafiltration permeation from whey (a) in terms of double enzyme catalysis whey protein cross‐linking coupling ultrafiltration including Laccases + transglutaminase (TG) (b), Tyrosinase + TG (c), TG + Tyrosinase (d) and TG + Peroxidase (e), respectively

To evaluate the whey filtration efficiency after enzyme catalysis protein cross‐linking, the EEM spectra of the ultrafiltration permeates were used to investigate the untreated whey and enzyme catalysis. Figure 3 presents the fluorescence EEM spectra of permeates from untreated whey and enzyme catalysis whey during ultrafiltration. Because β‐lactoglobulin and α‐lactalbumin contain tyrosine and tryptophan, the EEM spectra could show whether the permeation contains protein. Five samples including whey (a), laccases + TG (b), tyrosinase + TG (c), TG + tyrosinase (d), and TG + peroxidase (e) were chosen according to whey protein recovery rate.

As shown in Figure 3(a), three characteristic peaks, A (Ex/Em: 276 nm/335 nm), B (Ex/Em: 233 nm/340 nm), and C (Ex/Em: 375 nm/525 nm) were present in the fluorescence EEM spectrum of untreated whey UF permeate. It was known that peaks A and B were associated with proteins containing tryptophan and tyrosine (Svensson & Andersen, 2014). Peak C was associated with humic acid substances. These results verified the occurrence of protein‐like and humic‐like components in untreated whey UF permeate. When whey protein was catalyzed by tyrosinase + TG, the intensities of UF permeate at peaks A and B decreased to some extent as shown in Figure 3(c), indicating the partial rejection of protein‐like substances. When whey protein was catalyzed by laccase + TG in Figure 3(b) and TG + peroxidase in Figure 3(e), the intensities of UF permeate at peaks A and B slightly increased compared to those of the control, indicating that more protein‐like substances permeated. When the composite enzyme TG + tyrosinase was used to catalyze whey protein cross‐linking prior to UF, the permeation peak intensities were sharply reduced for protein‐like substances, as shown in Figure 3(d). The composite enzyme TG + tyrosinase during UF caused most of the whey protein to be rejected. Accordingly, the protein in the permeation decreased. Peak C existed in both untreated whey and enzyme‐treated whey UF permeates, which implied that the humic acid substances could not be rejected by the membrane under any conditions. Proper cross‐linking allowed easier filtration for the cross‐linked protein because of different surface charges and conformations under various catalysis conditions. These factors would affect the membrane and protein interactions and protein–protein interactions. In this study, the TG catalysis whey protein followed by tyrosinase (TG + tyrosinase) improved the ultrafiltration process.

### 3.4 Relationship between membrane resistance and the shape of proteins

**FIGURE 4:**
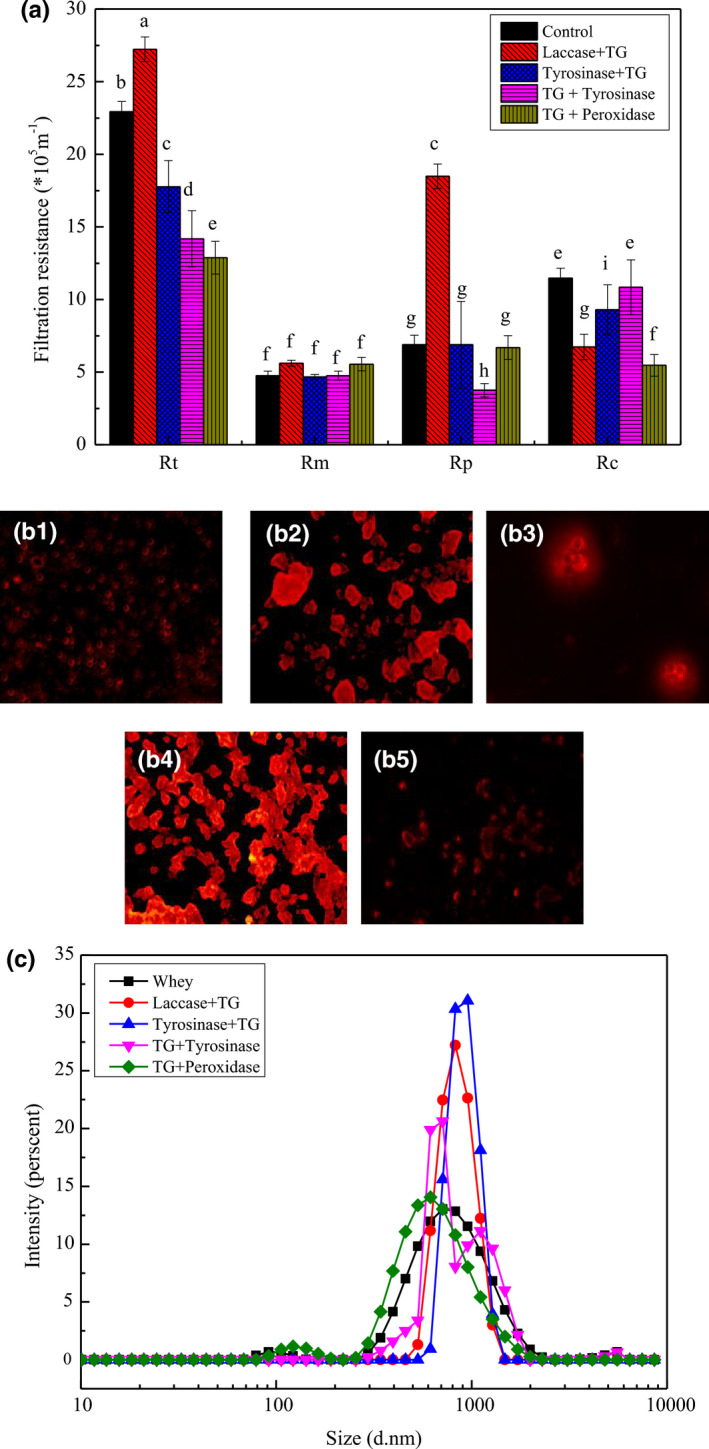
The relationship between membrane resistance and the shape of cross‐linked protein during ultrafiltration. The membrane resistance of different enzyme catalysis protein aggregation coupling ultrafiltration (a); Fluorescence microscope observation of particles morphology of aggregated proteins (b1) Whey, (b2) Laccase + transglutaminase (TG), (b3) Tyrosinase + TG, (b4) TG + Tyrosinase, and (b5) TG + Peroxidase, respectively

The total membrane resistance, pore blockage, and cake resistance were investigated at the end of filtration. As shown in Figure 4(a), the total membrane resistance (R_t_) of the laccase + TG sample was higher than that of the control. The other composite enzyme caused a decrease in the total membrane resistance. The membrane resistance reduction may have caused difficulty in filtration when the protein was cross‐linked under certain conditions. The total membrane resistance (R_t_) of the tyrosinase + TG sample was lower than that of the control. The size and shape determine the way particles stack and the cake's porous structure (Perini et al., 2021). The fluorescence microscopy results showed that the natural whey protein was spherical, as shown in Figure 4(b_1_), which is why the membrane pore blocking decreased with increasing in membrane filtration time. In Gao's study, the unique folding behavior resulted in higher penetration for the plate‐like shape particles (Gao et al., 2020). This indicated that the three‐dimensional and irregular shape was good for filtration. When the protein was catalyzed by the composite laccase + TG, the shape of the cross‐linked protein was similar to the chain structure as shown in Figure 4(b_2_). This structure increased the membrane pore blocking and reduced the cake resistance (*R*_c_) compared to the control. This finding also indicated that membrane pores were blocked by protein when ultrafiltration occurred after laccase + TG enzyme catalysis. Irregularly shaped particles such as branched carbon particles provided higher fluxes due to the high voidage cakes. More regularly shaped particles such as glass spheres resulted in lower fluxes (Connell et al., 1999). Additionally, the R_t_ of the laccase + TG sample was very high, as shown in Figure 4(a), and the relative flux was high, as shown in Figure 2. The relative flux of the laccase + TG catalytic protein was higher before 60 min and decreased as filtration time increased. More protein blocked the membrane pore or adhered to the membrane surface, thereby increasing the membrane total resistance at the end of the filtration. The *R_t_* and *R_p_* of the TG + tyrosinase sample were lower than that of the control. The *R_c_* value of the TG + tyrosinase sample was similar to that of the control. However, the cake layer of the control was stronger than that of the cross‐linking protein. The shape of the cross‐linking protein catalyzed by TG + tyrosinase was partially spherical and partially cylindrical, similar to the elongated shape shown in Figure 4(b_4_). The shape was similar to the crumby structure of tyrosinase + TG (b_4_) and TG + peroxidase (b_5_), which had lower filtration efficiency. In Bourcier's study, the cube and needle shapes exhibited a very porous cake and faster filtration compared to sphere and platelet shapes. This result means that the quickest filtration was obtained with these shapes. Furthermore, small proteins showed a higher resistance than large particles (Bourcier et al., 2016). This finding could explain why the relative flux increased after TG + tyrosinase catalysis whey protein cross‐linking. The increased size of whey protein could form loose cake on membrane surface which could help reject many small proteins, so the total whey protein rejection rate increased. As shown in Figure 3(c), the particle size of enzyme catalysis whey protein cross‐linking was increased compared to control (whey protein) except the decreased size of protein catalyzed by TG and peroxidase. For the intensity of TG + tyrosinase sample, there were two peaks distribution for the enzymatic aggregated protein. This size distribution may be effective for ultrafiltration. Furthermore, the protein cake formation on membrane surface was easy to clean compared to membrane pore blocking (Norazman et al., 2013). Furthermore, the membrane pore resistance of TG + tyrosinase sample was lowest of all, as shown in Figure 4(a). This result could explain why the TG + tyrosinase catalysis sample showed decreased membrane fouling. The membrane resistance decreased not only due to the increase in protein size but also because of shape changes.

### 3.5 Morphology of the membrane surface before and after filtration

**FIGURE 5:**
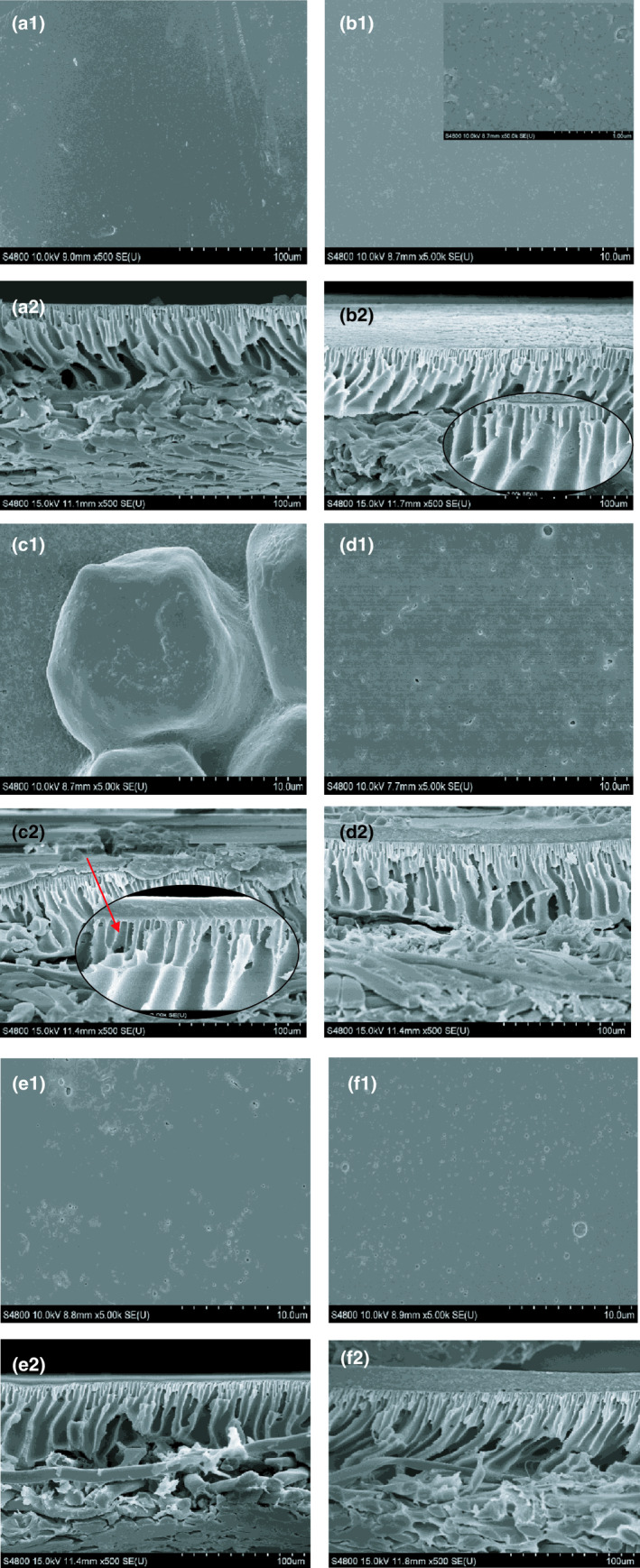
The SEM images of membrane surface (1) and pore (2), raw membrane (a1) and pore (a2), whey protein filtration without enzyme treatment (b1) and (b2), Laccase + TG (c1) and (c2), Tyrosinase + TG (d1) and (d2), TG + Tyrosinase (e1) and (e2), TG + Peroxidase (f1) and pore (f2)

The image of raw membrane before filtration is given in Figure 5(a1) and (a2). It can be seen that the ultrafiltration membrane surface was smooth and membrane pores were clean. After whey filtration, the membrane surface was covered by whey protein as shown in Figure 5(b1). The raw membrane surface layer was replaced by a dense layer formed by whey protein. The whey protein layer on the membrane surface formed the membrane cake resistance as shown in Figure 4(a1). At the same time, small proteins adhered to the membrane pore as shown in Figure 4(b2). As shown in Figure 5(c1), the membrane surface of laccase catalyzed whey protein cross‐linking followed by TG enzyme after filtration was covered by bigger protein aggregates which increased the total membrane resistance. However, the aggregates on membrane surface were easily removed by the feed during filtration and the membrane cake resistance was decreased compared to the control. Though some aggregates might be the result of a thick layer of foulant formed on the surface of the membrane layer that eventually induced bigger aggregate formation, the aggregates were easily washed by water or feed, which did not result in a dense cake layer on the membrane surface (Gebreyohannes et al., 2016). However, there were some proteins and small particles deposited in the membrane pores as shown in Figure 5(c2). Therefore, the membrane pore resistance was high for laccase and TG catalysis whey protein after filtration. For the membranes of tyrosinase and TG enzyme catalysis whey protein after filtration shown in Figure 5(d1), proteins deposited on the membrane surface formed a protein layer. There were some protein particles dispersed on the membrane surface, and the particle size was bigger than whey protein in Figure 5(a1). There were little proteins adhered on membrane pores which decreased the membrane pore blockage as shown in Figure 5(d2). As shown in Figure 5(e1), the membrane surface of TG and tyrosinase catalysis whey protein after filtration was covered by irregularly shaped proteins forming membrane cake resistance. The membrane surface of TG and peroxidase catalysis whey protein after filtration was covered by proteins in an uneven dispersion as shown in Figure 5(f1). Bigger aggregates were decreased compared to TG and tyrosinase catalyzed whey protein on the membrane surface in Figure 5(e1) for which the membrane cake resistance and total membrane resistance were lower than other conditions (Ding et al., 2019). As shown in Figure 5(f2), the membrane pore of TG and peroxidase catalysis whey protein after filtration had no significant protein adhesion. So the membrane cake resistance and pore blockage were lower than control. However, the protein recovery rate was lower than the sample of TG and tyrosinase as shown in Figure 1. This was mainly due to some proteins crossing into the membrane pore as shown in Figure 2(e).

### 3.6 Fourier‐transform infrared spectroscopy analysis of the membrane surface before and after filtration

**FIGURE 6:**
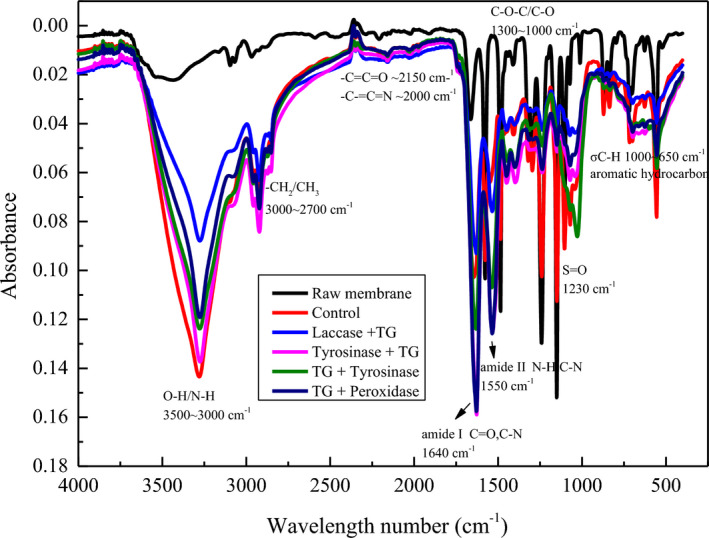
Fourier transform infrared spectroscopy (FTIR) spectra of raw membrane, whey protein filtration membrane without enzyme treatment (Control), Laccase + TG, Tyrosinase + TG, TG + Tyrosinase and TG + Peroxidase

The FTIR spectra for the raw UF membrane and the membrane fouled by proteins are shown in Figure 6 for comparison. The data were best for qualitative discussion and not for quantification; thus, no absorbance subtraction of fouled membrane from the virgin membrane was done (Liu et al., 2020). The higher broad peak at 3,250–3,500 cm^−1^ belonged to the stretching vibration of O‐H in the whey protein filtered membrane and corresponding peak in the enzyme‐catalyzed whey protein membrane after filtration membrane was comparatively increased. This was due to the membrane fouling of whey protein on the membrane surface. In particular, the peaks at 1,580 and 1,620 cm^−1^ were characteristic of PES membrane. At first glance, it appeared that most of the spectra were the same between the virgin of fouled membranes. Nonetheless, there was a remarkable increase in absorbance for the fouled membrane. The increased FTIR absorbance for fouled membranes might be because aromatic groups of the original membrane were now coated with nonaromatic or less aromatic substances. This might be the case, since whey protein molecules were the major source of fouling on the membrane surface. The slightly differences were the different enzyme absorbance peaks and the cross‐linked whey protein bonds. The appearance of bands in the range of 1,040–1,150 cm^−1^ can be assigned to the C=O stretching vibrations from whey protein. The amide I peak (C=O, C‐N) at 1,640 cm^−1^ and amide II peak at 1,550 cm^−1^ (N‐H, C‐N) were related to peptide bonds (CO‐NH). The intensity in the range of 1,040–1,150 cm^‐1^ was different for the sample of tyrosinase + TG and TG + tyrosinase. This indicated that the enzyme catalysis order was important for degree of protein polymerization. The increased broad peak at 710–500 cm^−1^ was mainly due to the benzene ring substitution zone. This means that the fouling membrane surface had aromatic amino acids which contained benzene rings. Some amino acids were from whey protein, and some were from the enzyme tyrosinase which came from the TG and tyrosinase catalysis whey protein cross‐linking with caffeic acid. Another band at 1,230 cm^−1^ related to the S=O bond in the sulfonate functional groups from PES membrane. The decrease in peak intensity at 1,230 cm^−1^ was mainly due to the fouling on membrane surface which covered the S=O bond groups of the PES membrane (Hoseinpour et al., 2018).

### 3.7 Interactions between proteins and membranes based on XDLVO theory

**FIGURE 7:**
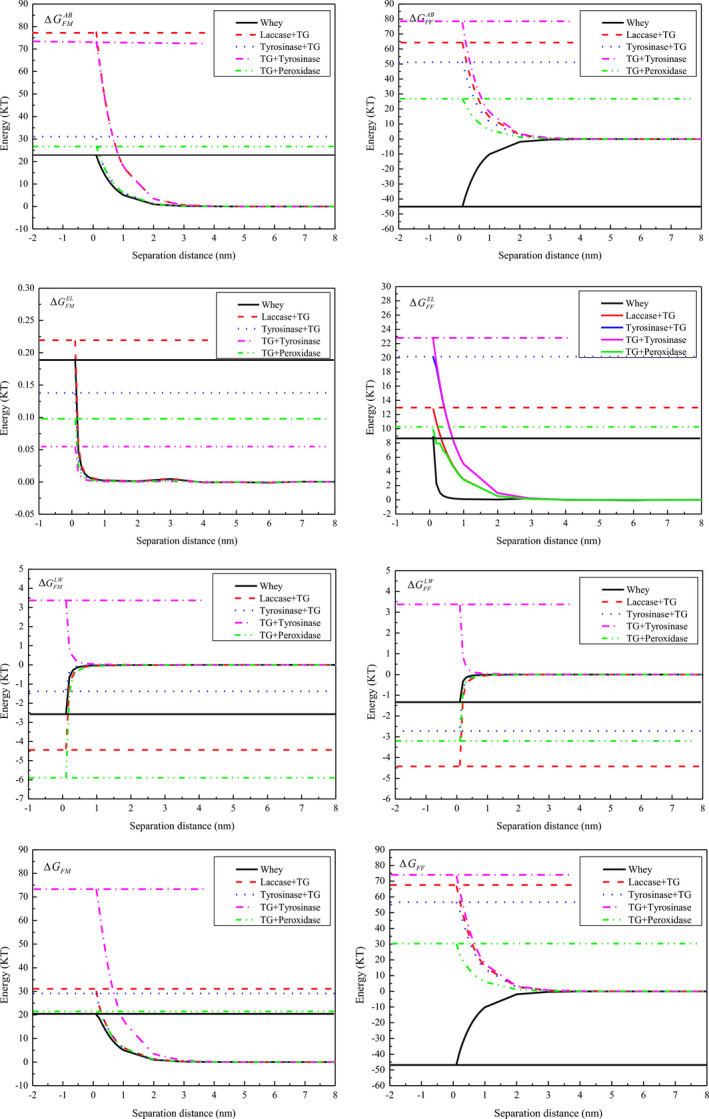
Interaction energy potentials between membrane surface and protein molecule interaction energy ($\Delta G_{\text{FM}}$) changes were given in the left hand column of panels (left hand column of panels; top to bottom: Lifshitz van der Waals force energy $\Delta G\frac{\text{LW}}{\text{FM}}$; Acid‐base interaction energy $\Delta G\frac{\text{AB}}{\text{FM}}$; Electrostatic interaction energy $\Delta G\frac{\text{EL}}{\text{FM}}$; Total interaction energy ($\Delta G_{\text{FM}}$) and the protein‐protein interaction energy ($\Delta G_{\text{FF}}$) changes are given in the right hand column (right hand column of panels; top to bottom: $\Delta G\frac{\text{LW}}{\text{FF}}$; $\Delta G\frac{\text{AB}}{\text{FF}}$; $\Delta G\frac{\text{EL}}{\text{FF}}$; $\Delta G_{\text{FF}}$)

The XDLVO theory can predict the interaction energies between the protein and the membrane surface (Tian et al., 2013). $\Delta G_{\text{FM}}$ represents the energy needed for the adhesion of the surfaces of the protein foulant (*F*) and membrane *(M*) when both are immersed in water. The protein–membrane interaction energy ($\Delta G_{\text{FM}}$) changes are given in the left‐hand column of panels, and the protein–protein interaction energy ($\Delta G_{\text{FF}}$) changes are given in the right‐hand column in Figure 7. ΔG was included in the Lifshitz van der Waals force energy ($\Delta G^{\text{LW}}$), electrostatic interaction energy ($\Delta G^{\text{EL}}$), and acid–base interaction energy ($\Delta G^{\text{AB}}$). ΔG < 0 and ΔG > 0 represent attractive and repulsive forces, respectively. As shown in Figure 7, the $\Delta G_{\text{FM}}^{\text{AB}}$ of the TG + tyrosinase catalysis whey protein cross‐linking was highest of all. The values of $\Delta G_{\text{FM}}^{\text{EL}}$ and $\Delta G_{\text{FM}}^{\text{LW}}$ were significantly lower than the value of $\Delta G_{\text{FM}}^{\text{AB}}$. Furthermore, only the TG + Tyrosinase cross‐linked protein showed repulsive Lifshitz van der Waals force energies. The positive values represented the degree of hydrophilicity, and negative values represented the degree of hydrophobicity (Chen et al., 2012). Apparently, the cross‐linked protein catalysis by TG followed by tyrosinase significantly increased the hydrophilic interactions between the protein and the PES membrane. The stronger repulsion energy between the cross‐linking protein and the membrane surface led to less accumulation of protein on the membrane surface, while the increase in the small EL interactions ($\Delta G_{\text{FM}}^{\text{EL}}$) between proteins and the membrane surface plays only a minor role. The contribution of AB interactions to the overall interaction energy is bigger than EL and LW. $\Delta G_{\text{FF}}^{\text{AB}}$ and $\Delta G_{\text{FF}}^{\text{LW}}$ of whey protein were negative, indicating the attractive interaction energy between whey proteins. However, the cross‐linked whey protein had positive values between proteins. From the AB interaction energies, conclusions can be drawn concerning the hydrophilic or hydrophobic properties of a surface. Surfaces with positive AB interaction energies are termed hydrophilic, as particle–water interactions are favored over polar particle–particle interactions. Hydrophobic surfaces have negative AB interaction energies, which (in the absence of other stabilizing forces) leads to particle aggregation (Kühnl et al., 2010). The cross‐linking protein catalysis by TG and tyrosinase had higher repulsive energy than that of the cross‐linking protein catalysis by laccase and TG. Therefore, total membrane filtration resistance (*R_t_*) was lower in the laccase + TG catalysis during UF, as shown in Figure 3(a). Therefore, TG and tyrosinase catalysis cross‐linking eased the membrane filtration process. The interaction energy estimation showed that double enzyme catalysis whey protein cross‐linking during UF made the repulsive energy increase between the proteins and the membrane. This finding explains the mechanism of the increase in relative flux and decreased membrane fouling.

## 4 CONCLUSION

The order of the double enzyme catalysis was found to be very important in determining the degree and filtration efficiency of whey protein aggregation. The TG catalysis whey protein followed by tyrosinase (TG + tyrosinase) could increase protein recovery rate which could reach 84% higher than that of the other conditions. The relative flux was highest of all in the initial 15 min and slightly decreased as filtration time increased. The shape of the protein had an effect on membrane resistance and relative flux by the fluorescence microscopy imaging. The shape of the cross‐linking protein catalyzed by TG + tyrosinase was partially spherical and partially cylindrical, similar to the elongated shape. Furthermore, the membrane surface of TG and tyrosinase catalysis whey protein after filtration was covered by irregularly shaped proteins forming membrane cake resistance from the SEM image. The bigger aggregates proteins forming loose cake on membrane surface could decrease membrane fouling and extension filtration time. Based on the extended XDLVO analysis, the repulsive energy interactions between the cross‐linked protein and membrane and between the cross‐linked proteins themselves increased. Therefore, TG followed by tyrosinase catalysis could increase the membrane filtration efficiency. This strategy can also be applied to other low concentration protein recycling fields.

## CONFLICT OF INTERESTS

The authors declare that they do not have any conflict of interest.

## ETHICAL APPROVAL

This study does not involve any human or animal testing.

## Data Availability

The data that support the findings of this study are available from the corresponding author upon reasonable request.
